# A qualitative exploration of experts’ views about multi-dimensional aspects of hookah smoking control in Iran

**DOI:** 10.1186/s12889-024-19139-9

**Published:** 2024-06-22

**Authors:** Sara Dadipoor, Azin Alavi, Hadi Eshaghi Sani Kakhaki, Nahid Shahabi, Zainab Kader

**Affiliations:** 1 Tobacco and Health Research Center, Hormozgan University of Medical Sciences, Bandar Abbas, Bandar Abbas, Iran; 2 Mother and Child Welfare Research Center, Hormozgan University of Medical Sciences, Bandar Abbas, Iran; 3 The Centre for Interdisciplinary Studies of Children, Families and Society, University of the Western Cape, Bellville, South Africa

**Keywords:** Hookah, Nicotine dependence, Smoking cessation, Qualitative study

## Abstract

**Background:**

The related literature has primarily addressed cigarette smoking control. It seems that researchers have failed to explore the determinants of hookah smoking (HS) control. In an attempt to fill this gap, the present study explores experts’ views about aspects of HS control in Bandar Abbas, a city in the south of Iran.

**Methods:**

The present qualitative study, conducted in 2022 and 2023, used a content analysis. To this aim, 30 experts in tobacco prevention and control were invited to participate in the research. Twenty seven accepted the invitation. In-depth, semi-structured, and face-to-face interviews were held with the experts. A purposive sampling was used and the data collection continued until data saturation. The interviews lasted between 18 and 65 min. MAXQDA 10.0 was used for data management and analysis.

**Results:**

The expert interviewees had a mean age of 44.77 ± 6.57 years and a mean work experience of 18.6 ± 6.8 years. A total number of six main categories were extracted from the data, including usin influential figures to control HS, controlling HS by alternative activities, changing beliefs and attitudes toward HS, taking administrative and regulatory measures, and facilitating HS cessation.

**Conclusion:**

This qualitative study explored the multifaceted ways people adopt to quit HS. Using influential figures to control hookah smoking, promoting alternative activities as a means of control, changing beliefs and attitudes, enforcing administrative regulations, and facilitating quit attempts all play an important role in tackling the prevalence of hookah smoking. These findings emphasize the importance of a comprehensive and multifaceted approach to integrate various interventions to effectively address hookah smoking behavior.

## Introduction

Hookah is a smoking device used in many countries and is also known as waterpipe, argileh, shisha, goza and narghile. In this device, smoke passes through water in a bowl, where it is cooled and filtered before being inhaled. Hookah is a traditional device for tobacco consumption [1], originating from the Middle East. Today, it is globally popular particularly among young adults and women [2, 3]. In the world, flavored tobacco and the absence of regulatory policies have led to the increased rate of hookah smoking (HS) [4]. As recently reported by WHO, tobacco consumption would account for 8 million cases of mortality worldwide on an annual basis [5]. As the research by Le et al. showed, current hookah smokers (HSs) had a 37% higher odds of mortality from all causes than non-smokers, while former HSs had a 39% higher odds of mortality from any cause than non-smokers [6].

According to a review article, most studies showed an increasing rate of HS between 2009 and 2016. This increase has ranged between 0.4 and 2.9% annually in East Mediterranean area and between 0.3% and 1% in Europe [7]. The prevalence of HS varies significantly across gender and region in the Middle East. In 2019, the prevalence among males and females was estimated to be 32.7% and 46.2%, respectively, in Lebanon, 13.4% and 7.8% in Jordan, and 18.0% and 7.9% in Palestine [8]. HS, especially among women, is becoming more and more socially acceptable as a normative behavior in the region [9]. In Iran, it is estimated that 82% of women who smoke tobacco use hookahs [10]. The overall prevalence of HS among Iranian women is reported to be 3.8–6.3% [11, 12]. However, there are large regional variations in HS in Iran. The prevalence of HS in women in the southern provinces such as Hormozgan is 9–10 times as high as other provinces [13]. In Bandar Abbas in Hormozgan, the prevalence is 15.1%, which is higher among women than men [14, 15]. The high prevalence of HS in Hormozgan can be due to the local culture, underestimated HS health risks, variety of jobs found in hookah cafes, and the lack of any tobacco control measures [16, 17].

As a complicated behavior, HS is influenced by many internal and external factors. Some are personal, yet others are interpersonal, social, political and organizational. Among these factors are positive attitude, underestimated health risks of HS, psychological and social gaps, physical and mental attachment to hookah, family issues, media advertisement, ease of access (availability) and the absence of prohibitory rules and poor monitoring and management [16, 18, 19]. Family support, social and psychological needs, family norms, control of external stimuli and political factors have been among the major factors involved in hookah cessation [20].

Although the control of effective factors in HS or hookah cessation can, to some extent, help prevent this unhealthy behavior, exploring the determinants of HS control can be particularly useful. The related literature has focused more on controlling cigarette smoking and attended less to all aspects of cigarette smoking prevention and control. Each study in the literature has only addressed one aspect of the matter [21–23]. Researchers have largely neglected the exploration of determinants of HS control. To the best of the present researchers’ knowledge, few qualitative or quantitative studies have been conducted on tobacco control strategies, especially about HS. Thus, it is essential to fill this gap in literature. The field experts’ comments need to be solicited. The present study explores the experts’ views of the aspects of HS control in Bandar Abbas, a city in the south of Iran.

## Materials and methods

### Study design

The present study employed a qualitative approach, and held in-depth, semi-structured, face to face interviews in Bandar Abbas, a city in the south of Iran in August 2022-June 2023.

### Setting

Of note is that, in Bandar Abbas city in Hormozgan, HS has cultural-historical roots. Tobacco use has run in this city for long. More particularly, hookahs have passed down from older generations to the younger. Hookahs are commonly used to entertain guests in ceremonies of joy and sorrow.

The prevailing culture in Bandar Abbas normalizes HS more than cigarette smoking. HS is very common in women’s get-togethers [24]. Also, the weather conditions and facilities of the city have made HS a recreational activity for the public, especially during seasonal economic recessions when people have more spare time [16]. Moreover, the influence of stakeholders in tobacco industry has further spread HS in Bandar Abbas and southern Iran [16].

### Participants

Initially, 30 experts in tobacco prevention and control were invited to participate in the study. Twenty-sevel experts accepted the invitation to participate. They had at least 5 years of work experience in controlling and preventing tobacco consumption. They had at least a bachelor’s degree of science to be included in the study.

#### Inclusion criteria

having academic qualification in the topic of interest.

#### Exclusion criterion

unwillingness to participate in the research.

### Interview

The interview guide contained two parts, one enquiring about demographic information such as age, and place of residence, and the other concerning the participants’ overt and covert beliefs about the HS. The interview guide was checked by a panel of five experts in smoking control and qualitative research methodology to decide wether it was appropriate for the study. Adaptations (based on participants’ feedback) were made to the guide after the first five interviews. Once the interview guide was adapted and finalized, the final version was used as the basis of all remaining interviews. The interviews lasted between 18 and 65 min.

Each interview began with four main questions in the interview guide. As the interview continued, follow-up questions were asked to get more details. Probe questions were asked when further exploration was needed. Table 1 contains a list of questions that were asked during the interview.

**Table 1:** Interview Guide

No	Question
1	In your opinion, what strategies can help people to cease HS?
2	What should others do to help people cease HS?
3	What do you suggest we can do to control HS in this city?
4	what should the government, NGO’s, etc. do to help people to cease HS?

### Data collection

The interviews were conducted by two researchers. Each interview took approximately one hour. The interviews were conducted at a time and place convenient for the participants. All interviews were held in a quiet place such as the expert’s work office, a private room at the research center, or a place preferred by the participant such as a park or coffee shop. The sampling method was purposive and snowball. After each interview, the interviewee was asked to suggest the next participant. The anti-tobacco consumption organization in Hormozgan Province was visited to find the first expert to interview. After making an appointment with the first expert, the time and place of the interview were set as the interviewee preferred. When the interview was done, the interviewee was asked to suggest a colleague for the next interview. Therefore, purposive and snowball sampling were used to include the experts. The data collection continued until data saturation.

### Rigor

The following attempts were made to increase the rigor of findings: (1) Sufficient time was spent on data collection (August 2022-June 2023); (2) To make sure of the accuracy of researchers’ interpretation of expert comments, the findings were made available to eight participants via random sampling. After receiving their feedback, minor changes were made to the data; (3). The data were provided to the 2nd and 4th authors who were expert in qualitative research. Their comments helped define and revise the categories and sub-categories. To ensure the confidentiality of findings, the categories, sub-categories and a sample coding were provided to two external experts with a robust confidentiality agreement. Comments made by these experts and the present researchers were in some cases contradictory. These contradictions were resolved through discussion and in reference to the initial interviews. Initially, a total of 7 main categories were identified from the data. Among these categories, there was a contradiction in the number of two classes. Following discussion and decision-making by the authors, two classes were merged into a single category named “Using influential figures to control HS.” Additionally, there was a discrepancy in naming the 3 sub-categories within the categories.

### Ethics approval and consent to participate

As for ethical considerations in this study, the procedure was approved by the Hormozgan University of Medical Sciences (#IR.HUMS.REC.1400.369). The purpose of study was revealed to all participants and they were ensured of the confidentiality of information they provided. All participants were required to sign an informed consent and were assured they could withdraw in any phase of research. All the research procedure was accordance with the relevant guidelines and regulations of research ethics.

### Data analysis

All interviews were audio-recorded and then transcribed. After a detailed initial textual analysis of each interview, the next interview was made. The interviews were reviewed independently line by line with an open coding approach to identify the underlying concepts in participants’ statements. When the analysis went on, the code and category extraction followed. The similarities and differences were found and distinguished from each other in terms of inherent features and dimensions. Finally, through comparing the categories, some sub-categories were merged and the main categories were finally formed. Researchers reviewed all the extracted codes in a meeting and discussed the categories and subcategories. They agreed on the majority of categories and subcategories, and only disagreed on a few cases, later solved by referring to the initial interviews and re-examining the codes. The extracted codes were processed in MAXQDA10.

## Results

Among the 30 experts in tobacco consumption invited to participate in the study, 27 entered the study. One refused to participate due to work obligations. The mean age of the expert interviewees was 44.77 ± 6.57 years. Their work experience ranged between 5 and 28 years with a mean value of 18.6 ± 6.8. Table 2 summarizes other relevant information.

**Table 2:** Experts’ demographic features (*n* = 27)

	Categories	Number	Percentage
Age (years)	30–40	7	25.93%
	40–50	12	44.44%
	> 50	8	29.63%
Educational level	Bachelor’s degree	6	22.22%
	Master’s degree	13	48.15%
	Ph.D.	8	29.63%
Marital status	Single	7	25.93%
	Ever married	20	74.07%
Work experience	5–15	5	18.52%
	15–25	17	62.96%
	> 25	5	18.52%
Work field	Municipality /governorate	5	18.52%
	Education	2	7.41%
	research	5	18.52%
	Health	6	22.22%
	Media	2	7.41%
	legislation	4	14.81%
	police	3	11.11%

Totally, six categories and 20 sub-categories emerged from the data analysis. The amount of data was very large, so we decided to focus only on determinants that had been less addressed in the literature. “Changing beliefs and attitudes toward HS” is not discussed, and only five categories and 17 subcategories are dicussed here (Table 3).

**Table 3:** Main categories, sub categories of determinants of HS control

Included/ Excluded	Categories	Sub-categories
Included in the study	Using influential figures to control HS	NGO’s Participation
		Family support
		Mass Media and social networks’ activities
		Peer education
		Popular figures and celebrities
	Controlling HS by alternative activities to HS market/trade	Innovative and creative entrepreneurship for sellers
	Controlling HS by alternatives to smoking as a habitual/recreational activity	Setting up recreational facilities
		Holding festivals and Joyful activities
	Taking administrative and regulatory measures	Anti-HS legislating and enforcing regulations
		Participatory administration
		Tax policies
		Citizens’ rights
		Segregation of HS places
		Setting limits
	Facilitating HS cessation	Founding HS cessation clinics
		Motivational services
		Mental health consultations
Excluded from the study	Changing beliefs and attitudes toward HS	Prohibiting any HS advertisement
		Showing HS as an abnormal behavior (in people with low self-esteem)
		Public education regarding HS adverse effects
		Use of religious beliefs against HS

The frequency and proportion of experts commenting on each subcategory are shown in Table 4. The subcategories are listed in descending order. Family support, with the frequency of 88.89%, is the most frequqnetly discussed topic by experts in the interviews.

**Table 4:** The proportion of experts in each subcategory (%)

Sub-categories	Frequency	Proportion (%)
Family support	24	88.89%
Peer education	17	62.96%
Mass Media and social networks’ activities	16	59.26%
Prohibiting any HS advertisement	16	59.26%
Public education regarding HS adverse effects	15	55.56%
Innovative and creative entrepreneurship for sellers	14	51.85%
Tax policies	14	51.85%
Holding festivals and Joyful activities	13	48.15%
Use of religious beliefs against HS	13	48.15%
NGO’s Participation	12	44.44%
Popular figures and celebrities	12	44.44%
Setting up recreational facilities	12	44.44%
Anti-HS legislating and enforcing regulations	11	40.74%
Participatory administration	10	37.04%
Setting limits	10	37.04%
Segregation of HS places	9	33.33%
Motivational services	9	33.33%
Mental health consultations	8	29.63%
Founding HS cessation clinics	7	25.93%
Showing HS as an abnormal behavior (in people with low self-esteem)	7	25.93%
Citizens’ rights	5	18.52%

### Using influential figures to control HS

“Using influential figures” showed to be a key determinant of HS control. This main category had several distinct sub-categories as addressed here.

#### Non-governmental organizations’ (NGOs) participation

As the majority of participants agreed, non-governmental organizations (NGOs) can bring many innovative ideas and potentials, and can significantly help prevent, control and cease HS if used besides executive governmental organizations. More public reliance on and better reception of NGOs can be one reason why NGOs should be involved in making the required preventive measures by the government. Below are some comments that the participants made on this category:

“Trying to incorporate NGOs can have dramatic effects because NGOs are created by people themselves. That is why the public trust them more, because they are seen as the link between people and the government. NGOs communic ate well with ordinary people”. (Female, 22 years of experience)

“NGOs have a great potential to help. If the government grants them a budget, they can manage it wisely. If NGOs have a well-defined goal, people welcome them and cooperate with them more”. (Female, 18 years of experience)

#### Family support

As the participants opined, family support and supervision can be a strong barrier to detrimental behaviors such as HS. Inadequate support can lead to deviation and improper decisions including one’s tendency towards HS.

“All factors affecting HS can be summarized as family support. If someone is both psychologically and spiritually supported by the family, s/he will hardly ever tend to smoke hookahs”. (Male, 20 years of experience)

#### Mass Media and social network activities

As the majority of participants agreed, forbidding any form of advertisement, direct or indirect, for hookahs in mass media can be an effective strategy to control and prevent HS. Introducing HS extensively as a health-threatening behavior in mass media can tremendously influence public belief and attitude. This is due to the trust people put in mass media. Below are some extracts from the participants’ accounts:

“Mass media has succeeded in annihilating certain unhealthy behaviors such as crack consumption. They highlighted the adverse effects and managed to create a deep fear of the drug in the public. Finally, the drug abuse was under control. HS can also be controlled in the same way”. (Female, 20 years of experience)

#### Peer education

Peer education was perceived by many participants as an effective strategy to control HS. Here is a sample extract from the interviews.

“I think if instructions are provided by peers, they are more effective because those emotions, attitudes and norms are better expressed. Teenagers listen carefully to peers and communicate with them better”. (Male, 5 years of experience)

#### Popular figures and celebrities

Many participants noted that the information provided or the kind of advice given by popular figures can significantly affect attitude to HS. These reliable sources can include family members, celebrities or popular football players among youngsters as well as clergymen who can talk against HS and discourage the negative behavior.

“If a celebrity begins to advertise against HS, that will help. Many followers will never want to smoke hookahs anymore or if they are already users, they may quit”. (Female, 16 years of experience)

### Controlling HS by alternative activities to HS market/trade

The majority of experts indicated that appropriate alternative activities to HS market/trade can act as an effective strategy to control HS. Below are several comments by the participants to support this idea.

#### Innovative and creative entrepreneurship for sellers

Most participants agreed that it was essential to find an alternative job for those who earned a living by selling hookahs. If their job was not replaced with a better one, they would never cease selling hookahs, and many socially adverse effects could follow.

“The government is supposed to use the least budget available to provide hookah sellers an appropriate job. For instance, the government can help them with interest-free loans. Or it can create a market where all these ex-hookah-sellers work and earn a living”. (Female, 18 years of experience)

### Controlling HS by alternatives to smoking as a habit/recreational activity

Controlling HS by alternatives to smoking as a habitual/recreational activity was another strategy suggested to control HS. As the interviewees commented, hookah can only be given up if it is replaced by a better choice. This will be explained here along with some extracts from interview content.

#### Setting up recreational facilities

Most participants agreed that extending recreational activities can significantly help control and reduce the rate of health-threatening behaviors such as HS. Unfortunately, Bandar Abbas is less equipped with recreational facilities than other cities. Thus, there is not a wide range of leisure activities to choose from.

“The more recreational facilities are provided for families, the less the probability of HS. Yet, these are largely absent here. Unfortunately, there is not even one good park or green space here”. (Male, 23 years of experience)

Reconstructing and renovating old urban areas (e.g., parks, gyms, pedestrian walks, biking lanes) which are red-spots for risky behaviors can be an effective strategy to prevent, control and cease HS.

#### Holding festivals and joyful activities

As most participants suggested, actively employing all existing sources can help prevent, control and cease HS. Instances of promising attempts are investment on young talents in art, music, theatre and the like, joyous celebrations in neighbourhoods, extending celebrations and festivals beyond official, indoor space to outdoor space and more specifically to neighbourhoods which can otherwise become a center for HS, and establishing the anti-hookah culture in such celebrations.

“I think if amusing programs were regularly planned in neighbourhoods, people would attend festivities or get-togethers instead of smoking hookahs. Such joyful events can provide a good chance for reminding people of the adverse effects of HS”. (Female, 23 years of experience)

### Taking administrative and regulatory measures

Participants agreed that the development of new rules and regulations was an effective strategy. These new rules should be preventive, controlling and inhibitive. As the interviewees admitted, there was currently no law against HS. If there was any, it was hardly put into practice. The following sub-categories provide more insights into this matter:

#### Anti-HS legislating and enforcing regulations

As many participants pinpointed, giving heavy fines for HS can, to a great extent, reduce the rate of the unhealthy behavior.

“Though many restaurants and coffee-shops are not allowed to sell hookahs, they break the rules and provide HS services. Immediately after they are fined, they get back to the same old habit. It is because there has been no severe legal prosecution. A minor fine does nothing to stop a high-income restaurant or coffee-shop owner selling hookahs”. (Female, 18 years of experience)

#### Participatory administration

As recurrently stated by several participants, Mutual cooperation of authoritative organizations can dramatically affect HS prevention, control and cessation.

“All those partly in charge of the program should join and start working together. They are to support each other and there should be a division of labor. Now, it is not the case because each organization is working on its own and not as a team. There is no follow-up. One or two organizations alone cannot do the whole thing”. (Male, 28 years of experience)

#### Tax policies

As suggested by many participants, putting higher taxes on hookah service providers such as coffee-shops can effectively prevent and control HS in society. They suggested hookah selling shops be divided into two, smoking and non-smoking. The former should pay higher taxes (3 or 4 fold).

“In many European countries, there are higher taxes on cigarettes and tobacco products. The same should go here. Coffee-shops that offer hookahs should pay taxes three times as high as others”. (Female, 23 years of experience)

“Municipal taxes should be 3–4 fold for coffee-shops that sell hookahs. These shops should pay taxes this year as they threaten citizens’ health. The next year, it is their choice whether they will continue selling hookahs or not” (Female, 19 years of experience).

#### Citizens’ rights

Most participants contended that raising the society’s awareness of citizen rights can largely change public view of HS. Air pollution follows from HS and when the public perceive themselves deprived of their right to have clean air, they learn to complain to those polluting the air. Here is a relevant comment:

“I think awareness of citizen rights can be a great help. We can change the public view. If a family passes by and looks down on me, that will be the end of me. No need to talk anymore! The mere silence means this is our right to enjoy clean air. Certainly, that will help”. (Female, 18 years of experience)

#### Segregation of HS places

Segregation of specific places for HS was another strategy that many participants suggested to prevent, control and cease HS. This category actually shows the necessity of making strict laws to take away hookahs from public places and confine them to enclosed spaces.

“If anyone who used to freely smoke hookahs at the beach or in parks is now forced to go indoors for smoking and knows s/he cannot smoke hookahs outdoors anymore, s/he might lose interest in smoking hookahs” (Male, 9 years of experience).

#### Setting limits

Effective strategies could include concentrating all hookah selling centers in one place, forbidding the sale of hookahs to those below 18, forbidding the sale of hookahs for 10 consecutive cases, keeping a distance of at least 100 m from schools, setting certain limits such as no food or drink served besides hookahs, reducing the attraction and facilities of hookah selling places, forbidding music, trees or plants around the area and other similar facilities to control and prevent HS. Others include being strict in giving the required work permissions to applicants.

“Shops that serve hookahs should be at least 100 meters away from schools. If not, they may be tempting to students, especially high school students who may tend to try different flavours when they find a shop nearby”. (Male, 10 years of experience)

“Certain limits should be set. For example, a hookah smoker should not be allowed to do so in parks or greeneries. Then, gradually, we can set stricter rules and say, for example, HSs are not allowed to watch TV and so on. No side dish should be allowed to be served with hookahs. This can tremendously cut down on the original attraction”. (Male, 28 years of experience)

### Facilitating HS cessation

Facilitating hookah cessation was another strategy suggested to control this tobacco product. It will be explained here along with extracts from interview content.

#### Founding HS cessation clinics

Trying to found tobacco cessation centers was mentioned as another back-up service to prevent, control and cease HS. The majority of smokers, when tired of the habit, look for places that can help them cease HS.

“If there are certain clinics exclusively established to help people cease HS, they can really help! People need to be notified at once and be encouraged to visit these clinics. The staff should be supportive experts that can attract people and teach them what to do in an interesting manner”. (Female, 21 years of experience)

“There exists no such a thing as an independent tobacco cessation clinic in our city! If such clinics are established and staffed with psychologists, physical educationalists and physicians, they will be a shekter to those tired of smoking”. (Male, 9 years of experience)

#### Motivational services

Most participants mentioned encouraging and motivating individuals or a mixture of motivational strategies could be an effective supportive strategy to control HS. Certain services such as travel ticket discount, concert ticket discount and gift cards for those who manage to cease HS can motivate them to continue the healthy behavior and encourage others to cease smoking. Allocating a budget to healthy entertainments such as cinema, concert, library and musical work can be another effective strategy in HS control. In other words, people can be provided with cultural activities at a low cost.

“If there is cultural subsidy for healthy reactions, for example, if they (i.e., the government) pay part of the cost for concerts, cinemas and gyms, everyone can enjoy healthy leisure at a low cost. The reason why almost everyone smokes hookahs is that it is a cheap amusement”. (Female, 17 years of experience)

Participants also believed that hookahs could not be taken away from consumers or salespeople unless they were replaced by appropriate hobbies.

“Obligation is not going to work! There should be some rewards. When something is taken away from someone, it needs to be replaced with something better. If you only think of HS as a hobby, you should begin to think what other hobbies can replace it. Even the salespeople should be provided with an alternative job”. (Female, 19 years of experience)

#### Mental health consultations

Many participants mentioned that mental health consultation can facilitate HS cessation. Supportive acts can include stress management through regular screening programs for mental health, active education on life skills from early childhood that can help people learn to react appropriately to stress, anger, temptation and learn to reject indecent suggestions made by peers. Another supportive service can be the establishment of centers to provide free face-to-face or on-call psychological services around the day. See the following comment.

“Most people find themselves smoking hookahs to escape stress. So, if such mental problems as stress are controlled from school days and even earlier from pre-school, what later leads to HS may be prevented”. (Male, 21 years of experience)

Concerning free psychological consultations, a participant quoted:

“If distressed families could refer to an advisor for help and be appropriately supported, they would for sure not have to retreat to HS to lower their stress. The advisor needs to be available and ready to help either face to face or on phone. Such advisors need to be supported by the executives” (Male, 26 years of experience).

## Discussion

The present research is pioneering in employing a qualitative content analysis to explore the determinants of HS control.

The interviewees believed that involving NGOs is a key strategy for HS control. Different NGOs, such as the Iranian Anti-Tobacco Association, are actively involved in tobacco control initiatives in Iran, with a focus on public health and environmental protection [25, 26]. The Iranian government, through the National Tobacco Control Headquarters supported by the government and monitored by the Ministry of Health and Medical Education, cooperates with relevant ministries, authorities, and NGOs [27]. The National Tobacco Free Initiative Committee (NTFIC) has actively cooperated and transferred information between the government and NGOs to speed up tobacco control endeavors in Iran [28]. Thee have been similar efforts in other countries like Romania and Pakistan, where NGOs actively help control tobacco use in joint efforts with national and international parties, and encourage the involvement of different organizations [29, 30]. In this regard, one study in India by Mondal et al. revealed that NGOs played a major role in tobacco control measures around the world. They acted effectively in raising the victims’ awareness and rehabilitating them by constantly supporting them in controlling this unhealthy behavior [31]. Therefore, it is suggested to use the capacity of NGOs in knowledge sharing and extending the culture further and allocating national budgets for its implementation.

Family support and supervision were found as another key strategy for HS control, according to the interviewees. This finding was also confirmed by other studies on family support which found it as an important factor in reducing the rate of HS [20, 32, 33]. Dana et al. studied adolescents in 42 countries and examined the long-term impact of family activities on adolescent smoking behavior in the United States. This study pinpointed the significant role of family support and supervision in reducing the rate of smoking among adolescents [34]. Family support can play a vital role in shaping attitudes and behaviors that help start and continue hookah use. Family support, especially during adolescence, has a continuous effect on reducing the risk of adolescent smoking [35]. Family support seems to play an important role in the tendency and desire to quit smoking When facing a challenge or stressor, others’ social support in an informal environment can help the adolescent cope with problems and stress. As a result, s/he will have a greater ability to manage the challenge or stressor, thus promoting supportive and close relationships. Fostering a supportive family environment and involving family members in cessation interventions can significantly contribute to lower rates of smoking and a healthier lifestyle.

The interviewees viewed mass media as another influential strategy to control HS. The use of appropriate health-promoting messages or motivational services is critical in supporting smoking cessation efforts [36]. It seems that mass media could advertise more effectively to tackle the issue at hand because people tend to trust them more; thus, acquiring information from these reliable sources can deeply influence their belief. Iran Ministry of Health has cooperated with relevant agencies to initiate a wide range of anti-tobacco mass media campaigns. These campaigns have mainly dealt with hookah consumption, youth, and females, and aimed to raise public awareness of the threats of tobacco consumption [27]. A relevant study among adults in the United States showed that mass media advertisements were positively correlated with the reduced rate of tobacco consumption [37]. Similarly, another study showed that mass media campaigns were considered a key strategy to reduce the rate of tobacco consumption among youngsters [38]. Mass media campaigns have been recognized as a powerful means of reducing tobacco consumption, especially among youngsters [39]. Mass media can be used for effective messaging in public health and for behavior change.

As the experts commented, peer education is another useful strategy for HS control. Peer education involves empowering community members to induce positive health changes within their peer group as a method of health promotion [40]. In an interventional study in Turkey among high school students, peer education was considered an effective method of changing tobacco smoking behavior [41]. The interactive nature of peer education makes it an important complement to HS control and other health promotion measures. Support groups, including peers, can play a low-cost but effective role in controlling unhealthy behaviors, such as HS. Peers understand each other better and accept health advice better from friends. Peer support groups also provide an opportunity to share experiences and eliminate the unhealthy behavior.

Information provision by popular figures and celebrities was another factor perceived by the interviewed experts as effective in controlling the above-mentioned unhealthy behavior. A relevant study in Iran among students of University of medical sciences showed that the advice from influential figures is an important factor in quitting smoking and reducing HS [42]. Celebrities often significantly influence their fans and followers, and their behaviors can shape social norms and perceptions [43]. This influence can be used to internalize cessation and reduction of smoking. Also, the engagement of celebrities in HS can normalize the behavior and create a perception of social acceptance. Targeting influential figures to promote healthy behaviors and discourage unhealthy behaviors can be an effective strategy to control the spread of HS and other unhealthy habits.

Another strategy suggested by the interviewees was alternative activities to HS market/ trade. One such alternative service was ‘innovative and creative entrepreneurship’ which involved finding an appropriate job to replace hookah sellers’ job. The rate of HS was higher in low- to average-income countries than high-income countries [44]. It appeared that economic pressures and lack of appropriate job opportunities led people to sell hookahs or offer hookah services. Hookah marketing has been probably considered an employment issue for low-income families with no better job opportunities. Local authorities ares suggested to provide special facilities to sellers to land suitable new jobs and reduce the sale of and access to tobacco products. Providing alternative economic opportunities, particularly through entrepreneurship and job creation programs, could be an effective strategy to control hookah use. To this aim, the underlying economic factors that lead people to hookah-related activities should be considered.

As the interviewed experts believed, another alternative strategy to smoking was the provision of recreational facilities. It seems that adding to the number of gyms and sport facilities in slums can significantly help prevent and control tobacco consumption. Some related Iranian research pointed out the lack of recreational facilities in Iran as an underlying reason for HS [45, 46]. Arguably, Bandar Abbas, as the main city in Hormozgan Province, lacks proper public recreational facilities such as amusement parks. In this city, the only public entertainment is spending time on the beach. Since the beach and surrounding areas do not have any entertainment facilities for different age groups, many opportunists seize the chance to sell and rent hookahs, therefore, many people smoke hookahs as a leisure. Authorities are suggested to consider recreation seriously and act effectively to renova te urban space to better control and cease HS.

From the viewpoint of the interviewed experts, Organizing festivals and joyful activitieswas identified as another strategy for controlling HS. This idea was supported by an Iranian study mong high school students that revealed that non-HSs achieved a higher happiness score than HSs [47]. Using all the existing capacities of the society can increase pleasurable activities of all members of society. Furthermore, it can be assumed that those who often experience a high level of happiness have fewer emotional and behavioral problems. These people would therefore be less likely to orientate towards HS. Festivals and joyful events may provide a social context in which HS is more common and can probably lead to increased consumption. Essentially, there is a need for national policies to create appropriate opportunities for people to show happiness.

There is also a need for ‘formulating regulations’ which can significantly help tackle the problem. One such rule/regulation can be heavy fines. As similar research on youngsters and adolescents showed, fining children and teenagers for carrying any form of tobacco product managed to reduce the rate of tobacco consumption to a large extent [48, 49]. Another study on reduced HS in youngsters in the United States showed that the anti-tobacco rule is mainly implemented for cigarettes and no strict rule has been set or implemented for hookahs [50]. It is noteworthy that while fines have been effective in reducing tobacco consumption, there is a lack of strict rules against HS in some regions. Thus, prohibitory rules and strict regulations, such as heavy fines, can be an effective way to prevent and control tobacco consumption, particularly HS, in Iran.

As the expert interviewees agreed, to control HS effectively, a participatory approach is needed to involve all relevant organizations. If the existing organizations in charge of HS control share duties and cooperate with each other, they can better manage to prevent and control the unhealthy behavior. Some research on proven strategies for smoking cessation showed that to challenge tobacco control, all organizations involved should act cooperatively and interdependently [51]. Probably, non-cooperative policies that the government makes were actively involved in HS control. Evidently, policymakers do not include the viewpoints of lower-ranking forces in HS control policymaking. If the comments made by lower-ranking forces or even smokers themselves are included, there will be better chances of compliance with rules and plans. Thus, policymakers are strongly recommended to take the advice by lower-ranking forces into account in decision making.

As the experts suggested, increasing tobacco taxes and prices is an effective measure for HS control. Increasing taxes, in a relevant work of research, managed to significantly lower the rate of smoking cigarettes [21]. A study by Hu, Mao, Shi, and Chen (2016) emphasized that increasing taxes is the easiest and most economical way to control tobacco consumption in China [52]. Higher taxes are followed by less demand in the market. Arguably, multifold taxation on coffee shops selling hookahs compared to others will reduce the profit of selling hookahs, which will be demotivating for sellers, and can reduce the supply of hookahs. Thus, it is expected that increasing taxes can reduce or correct the pattern of HS.

‘Familiarization of society with citizen rights’ was another effective strategy to control HS.

This factor shows that society’s awareness and understanding of individual rights can affect HS-related behaviors. When citizens get to know their rights and the consequences of HS, it can lead to more responsible and controlled HS behavior. A work of research revealed that a tobacco-free generation corresponds to citizen rights [53]. Katz (2005) showed that any attempt to control secondary tobacco smoke should be focused on individual rights. If people know it is their right to enjoy clean air, when they see others (HSs) depriving them of this right, they will react. This would not only affect their own belief but also that of the smoker. The latter needs to be more cautious as others can easily begin to complain. Therefore, this factor should not be neglected in controlling this unhealthy behavior.

The factor ‘Segregation of HS places’ was mentioned by the interviewees too as an effective strategy for HS control (HS). This approach involves creating designated areas or spaces specifically for hookah smoking, separate from other public areas. A systematic review revealed that segregating HS places can play a key role in controlling HS [19]. Another similar study showed that developing an anti-smoking rule in public places and implementing it carefully can lower the mean rate of smoking for about 4–10%. Thus, many people might cease smoking [54]. If HS is confined to particular places and banned in public space, it can help control HS effectively.

The expert interviewees believed that setting certain limits on the availability and purchase of hookahs can be an innovative rule to prevent, control or cease tobacco altogether. It appears that hookahs are more accessible to the public than other tobacco products. A body of research in Iran and Unites States point to the extensive and facile access to hookahs as a main reason for the high prevalence of HS [55, 56]. Overall, tobacco use seems to be significantly lower in cities with strict rules than in cities without any strict restrictive rule and regulation. Making prohibitory rules and eliminating the positive attitude and increasing the socially negative attitude to HS can significantly help reduce access to hookahs.

The interviewed experts suggested that establishing tobacco cessation clinics (TCCs) was another strategy to control HS. A study showed that TCC was capable of satisfying tobacco smokers’ needs and managed to stop hookah cessation. By providing effective educational interventions, these clinics manage to help smokers stop smoking cigarettes [57]. TCCs can meet the needs of HSs and provide effective educational interventions to help them quit. By providing exclusive cessation services to hookah users, TCCs can be as effective in HS cessation as in cigarette smoking [58, 59]. The existence of specialized smoking cessation clinics can point to the seriousness of this matter and encourage people to think about the adverse effects of HS. Therefore, building dedicated smoking cessation clinics for HS can be a great help for people who intend to quit hookahs.

The expert interviewees believed that providing motivational services was a strategy to control HS. A study at a Russian smoking-cessation center showed that individuals who were highly motivated to quit smoking had a success rate four times as high as those with lower motivation levels [58]. Providing appropriate motivational services, such as financial incentives, to individuals who have quit or intend to quit HS can effectively encourage and support their healthy behavior. A specific motivational service was suggested to be the provision of a cultural subsidy to address the affordability of hookah smoking in social settings. Roskin, Roskin and Aveyard (2009) reported that the low cost of HS among group amusements was a main reason for smoking hookahs [59]. By submitting a budget for cultural subsidies to increase healthy recreational activities, authorities can take effective measures to control this unhealthy behavior and encourage individuals to show healthier behaviors.

‘Mental health consultation’ was another strategies of HS control, as the interviewees suggested. A study of Armenian population in Tehran showed that a significant proportion of respondents raised the issue of frustration and psychological/spiritual problems at the outset of the unhealthy behavior of drug abuse [60]. Similarly, psychological needs and gaps were mentioned as the major reasons for HS [45]. It can be argued that people with insufficient problem-solving skills or failed self-assertion among friends turn to hookahs when feeling unhappy or lonely. Providing mental health counseling can help address the psychological aspects of HS and contribute to effective control measures. It proves the importance of mental health interventions as comprehensive strategies to prevent and reduce HS behaviors.

### Strengths, limitations and suggestions for further research

There were certain limitations in the present research. As in all types of qualitative research, the researcher’s own beliefs and perceptions could have affected the procedures from conceptualization to communication with participants and data interpretation [61]. Though in the present research, exploratory heuristics was used in data analysis to directly extract the categories and subcategorise from the data, it was possible that the interview questions did not cover all effective factors in HS. To compensate for this, the interviews continued until data saturation. Despite the above-mentioned limitations, there were several strengths too. The expert participants were selected from among the most knowledgeable in this area, with the benefit of proving realistic information for HS control. Further research is needed to explore these strategies in more extensive areas and from all demographic groups so that we can have access to comprehensive data about the effective strategies to prevent and cease HS.

### Implications

To the present researchers’ best knowledge, no study has been conducted to date to determine effective factors in HS control. The present findings can significantly fill the existing gap in the literature. Also, in future, these findings can form the basis of comparative studies. Finally, the present findings can guide policy makers to develop the necessary standards and guidelines to make effective plans and interventions to better control HS.

## Conclusion

This qualitative study explored the multifaceted ways people adopt to quit HS. Using influential figures to control hookah smoking, promoting alternative activities as a means of control, changing beliefs and attitudes, enforcing administrative regulations, and facilitating quit attempts all play an important role in tackling the prevalence of hookah smoking. These findings emphasize the importance of a comprehensive and multifaceted approach to integrate various interventions to effectively address hookah smoking behavior. Moving forward, targeted interventions based on these categories can significantly help reduce the prevalence of hookah smoking and promote healthy lifestyles among individuals.

## Data Availability

The datasets generated during and/or analyzed during the current study are available from the corresponding author on reasonable request.

## Abbreviations

HS: Hookah smoking
HSs: Hookah smokers
NGO: Non-governmental organization
